# 

**Published:** 1981

**Authors:** 


### Contents

3 **Editorial**

4 **Defining the Medical Profession in Mid-Nineteenth Century Bristol**
P. S. Brown

8 **Carotid Surgery in a District General Hospital**
John Fairgrieve

12 **St. Peter's Hospice, the evolution of terminal care for cancer patients in Bristol**
H. K. Bourns

15 **A visit to Poland**
John Cosh

19 **South West Orthopaedic Club**
Abstracts of papers given at the meeting in Truro on 2nd May 1981

23 **Book Review**
The Bristol Royal Infirmary 1904-74

24 **Examination Results**

